# Aloe-Emodin Induces Mitochondrial Dysfunction and Pyroptosis by Activation of the Caspase-9/3/Gasdermin E Axis in HeLa Cells

**DOI:** 10.3389/fphar.2022.854526

**Published:** 2022-05-17

**Authors:** Tonghui Li, Liuliu Shi, Wenqiang Liu, Xuhao Hu, Yuanjian Hui, Maojun Di, Shen Xue, Yan Zheng, Mengjuan Yao, Chen Li, Kun Meng

**Affiliations:** ^1^ Department of General Surgery, Affiliated Taihe Hospital, Institute of Infection and Immunity, Hubei University of Medicine, Shiyan, China; ^2^ Laboratory of Medicinal Plant, Hubei Key Laboratory of Embryonic Stem Cell Research, School of Basic Medicine, Institute of Basic Medical Sciences, Biomedical Research Institute, Hubei University of Medicine, Shiyan, China; ^3^ Department of Obstetrics and Gynecology, Affiliated Dongfeng Hospital, Hubei University of Medicine, Shiyan, China; ^4^ Department of Pharmacy, Hubei Aerospace Hospital, Xiaogan, China; ^5^ School of Public Health, Hubei University of Medicine, Shiyan, China

**Keywords:** aloe-emodin, mitochondrial dysfunction, bax-caspase-9-caspase-3 axis, gasdermin E, pyroptosis, transcriptome analyses

## Abstract

Aloe-emodin (1,8-dihydroxy-3-hydroxymethyl-anthraquinone), derived from some Chinese edible medicinal herbs*,* exerts a potential anticancer activity on various cancer cells, making it a drug candidate for cancer therapy. Yet, the role of aloe-emodin in pyroptosis, a new type of cell death, is uncharacterized. In this study, we explored the molecular mechanisms of aloe-emodin-triggered pyroptosis. Aloe-emodin inhibited proliferation and migration and triggered caspase-dependent cell death of HeLa cells in a dose-dependent manner. Aloe-emodin caused mitochondrial dysfunction and induced pyroptosis by activating the caspase-9/3/GSDME axis. Transcriptional analysis showed extensive changes in gene expressions in cellular pathways, including MAPK, p53, and PI3K-Akt pathways when treated with aloe-emodin. This study not only identified a novel role of aloe-emodin in pyroptotic cell death, but also performed a systematical genome-wide analysis of cellular pathways responding to aloe-emodin, providing a theoretical basis for applying anthraquinone derivatives in the treatment of GSDME-expressing cancers.

## Introduction

Pyroptosis, a newly recognized regulated cell death (RCD), is characterized by cell swelling and bubble-like morphology, which is different from apoptosis (Fang et al., 2020). Pyroptosis was initially recognized in immune cells as a general inflammation response against bacterial infection in the field of inflammatory diseases (Bergsbaken et al., 2009). In this case, gasdermin D (GSDMD) was cleaved by caspase-1/4/5/11, and its produced N-terminal fragment mediates the formation of the pores on the plasma membrane (Shi et al., 2015; Man et al., 2017). Activation of caspase-3 by chemotherapy drug could also induce pyroptosis by gasdermin E (GSDME) cleavage. The produced N-terminal fragment of GSDME causes pyroptosis by translocating to the plasma membrane (Jiang et al., 2020). *Gsdme* knock-out caused resistance to chemotherapeutic drugs in specific cancer cells and reduced chemotherapy-induced tissue damage in mice (Wang et al., 2017). Like chemotherapy drugs, natural products from Chinese medicinal plants possess potent anti-tumor activity, increase chemotherapy sensitivity and reduce its adverse effects, which supplements conventional chemotherapy anti-cancer drugs (Talib et al., 2021). The anti-cancer function of pyroptosis by chemotherapy drugs is rather well reported, but the effects of the active ingredient of the Chinese herbal medicines on pyroptosis are largely unknown.

Aloe-emodin (AE) is one of the anthraquinones compounds derived from traditional Chinese medicinal plants, such as *Rheum palmatum* L., *Aloe vera* (L.) Burm. f., and *Polygonum cuspidatum* Willd. ex Spreng.(Wang et al., 2008; Mandrioli et al., 2011; Wu Z. et al., 2019). Emerging studies are focusing on the anti-cancer properties of this compound. AE induces cell cycle arrest and triggers cell death in various cancer cells, and also increases the cellular sensitivity to chemotherapeuticagents (Chen et al., 2004; Chihara et al., 2015; Sanders et al., 2018). Caspase activation may also lead to GSDMs-mediated pyroptosis. Still, the effects of *Aloe Vera* or AE on pyroptosis in cancer cells have not been reported.

Here, we demonstrated that aloe-emodin triggers cell death through GSDME-dependent pyroptosis in HeLa cells. AE treatment induces mitochondrial dysfunction, leading to ROS production, cytosol release of cytochrome c, mitochondrial translocation of Bax and AIF, caspase-9 activation, and GSDME cleavage by active caspase-3. Furthermore, transcriptomic analyses show the potential cellular pathways upon AE treatment. Thus, our study reveals a novel role of AE in cancer cell pyroptotic death and provides a systematically transcriptional analysis of pathways and cell responses in HeLa cells. Collectively, our data provide a theoretical basis for applying anthraquinone derivatives in the treatment of GSDME-expressing cancers.

## Materials and Methods

### Antibodies and Reagents

Antibodies for GSDMD (ab209845) and GSDME (ab225893) were purchased from Abcam. Antibodies for Bcl-2 (60178-I-IG), Bax (50599-2-IG), and AIF (17984-I-AP) were purchased from Proteintech. Antibody for *α*-tubulin (T5168) was from Sigma-Aldrich. Antibodies for caspase-1 (98033), caspase-3 (9662S), caspase-8 (4790S), caspase-9 (9502S), cytochrome *c* (12963S), Bid (2002S) and Tom20 (42406S) were from Cell Signaling Technology.

Reagents were purchased as follows: anthraquinone derivations (Chrysophanol (AB0838), Emodin (AB0722), Aurantio-obtusin (AB0387), Physcion (AB0706), Aloe-emodin (AB0839), and Rhein (AB0861)) from alfabiotech, China. The purity of these anthraquinone derivations was confirmed to be 95%–99%, according to the manufacturer’s instructions. *N*-acetylcysteine (NAC) was purchased from Sigma-Aldrich (A0737). Anthraquinone derivations were dissolved in DMSO and diluted with fresh culture medium to the indicated concentrations. Caspase-3/7 substrate Ac-DEVD-AFC (A0466) and caspase inhibitor z-VAD-FMK (C2105) from Sigma-Aldrich; protease inhibitor cocktail tablets (04693132001) from Roche; CytoTox 96 Non-Radio cytotoxicity assay kit (G1780) and CellTiter-Glo^®^ 2.0 Cell Viability Assay (G9214) from Promega; FITC annexin V apoptosis kit I (556547) from BD Biosciences; Cell Counting Kit-8 (C0037), mitochondrial membrane potential assay JC-1 kit (C2006), GSH assay kit (S0053), SOD assay kit with NBT (S0109) and ROS assay kit (S0033S) from Beyotime.

### Cell Culture and Treatments

293T, HeLa, SW480, HT29, MCF7, and A375 cells were maintained in our laboratory and were cultured in DMEM (HyClone) supplemented with 10% fetal bovine serum (FBS) (Gibco), 2 mM l-glutamine, 100 U mL^−1^ penicillin, and 100 μg ml^−1^ streptomycin in a saturated humidity incubator containing 5% CO_2_ at 37°C.

For anthraquinone derivatives or aloe-emodin treatments, cells were grown to approximately 80% confluence, when the medium was replaced with a fresh medium containing indicated drugs and cultured for an indicated time. To inhibit caspase cascade, pan-caspase inhibitor z-VAD-FMK was pre-added into cells for 2 h. The concentrations of the drugs used were mentioned in the figure or figure legends.

### Cell Viability Assay

Cell proliferation was measured using a CCK-8 kit. Briefly, HeLa cells were cultured in 96-well plates overnight until the cell density reached ∼80% confluence, and then replaced with a fresh medium containing AE (0, 25, 50 μM) or cisplatin (5 μg/ml) for the indicated times. Then, the cells were added with 10 μl CCK-8 and incubated for another 2 h at 37°C before measuring the absorbance at 450 nm using a microplate reader. For ATP and LDH release assays, cells were treated with the indicated concentrations of AE for 24 h. Then, cells were subjected to ATP assay, and the supernatant was used for LDH activity according to the manufacturer’s instructions. All studies were performed in at least biological triplicates. The 50% cytotoxic concentration (CC50) was calculated by the GraphPad Prism 9.0 software.

### Wound Scratch Assay

Cell migration was evaluated using a wound scratch assay. HeLa cells were cultured on 6-well plates to ∼80% confluence. The wound was created by scratching a straight line on the monolayer cell with sterile pipette tips. After twice washing with PBS, cells were replaced with a serum-free culture medium in the absence or presence of 1 μM AE. Cell wound photos were taken with a light microscope at the indicated time post-AE-treatment. The wound width and the closure rate were calculated by ImageJ software.

### Cell Transfection

Transient transfection was performed using Jetprime (Polyplus) reagents following the manufacturer’s instructions. For over-expression, 293T cells were transfected with the pCS2-Flag-GSDME plasmid for 18 h, then subjected to AE or DMSO treatment for another 24 h, and analyzed as indicated. For siRNA knockdown, 200 pmol of siRNAs were transfected into 2×10^6^ HeLa cells. 48 h later, transfected cells were treated with 50 μM AE and anal as indicated. Sense sequences for the siRNAs used are as follows: *gsdme* 1#:yzed 5′-GGT​GAC​CTG​ATT​GCA​GTA​T-3′, *gsdme* 2#: 5′-GCA​GCA​AGC​AGC​TGT​TTA​T-3′, *gsdme* 3#: 5′-GGA​TTG​TGC​AGC​GCT​TGT​T-3′, and negative control (NC): 5′-TTC​TCC​GAA​CGT​GTC​ACG​T-3’.

### Caspase Assay

Caspase activities were assayed as described (Meng et al., 2016). Briefly, cell lysates were mixed with 20 μM Ac-DEVD-AFC in a Na-Citrate buffer (50 mM Tris-HCl, pH 7.4, 1 M Na-Citrate, 10 mM DTT, and 0.05% CHAPS), and incubated at 37°C for 30 min. Fluorescence intensities at λExc/λEm≈405/510 nm were measured every 5 min for 1 h at 37°C. Data were collected and analyzed using Graphpad Prism 9.0 software.

### Mitochondrial Membrane Potential (*ΔΨ*m) Measurement

*ΔΨ*m was measured using the mitochondrial membrane potential assay kit with JC-1. After drug treatment, cells were washed with PBS twice and replaced with a fresh cell culture medium. Cells were added with JC-1 dye working solution, mixed thoroughly, and then incubated for 15 min at 37°C in the incubator. Cells were observed under a fluorescence microscope after removing the supernatant and twice washing with JC-1 staining buffer. JC-1 accumulates in the matrix of mitochondria to form polymers (aggregates) when *ΔΨ*m is high, which can produce red fluorescence. When *ΔΨ*m is depleted, JC-1 cannot aggregate in the matrix of mitochondria. At this time, JC-1 is a monomer and can produce green fluorescence. In this way, the change of mitochondrial membrane potential can be measured through fluorescence color change (Reers et al., 1995). CCCP (carbonyl cyanide m-chlorophenyl hydrazone) was set as the positive control.

### Flow Cytometry Analysis

Adherent HeLa cells were washed with PBS, digested with trypsin, and collected by centrifugation. Then, cells were mixed with annexin V-FITC and PI staining working solution gently, incubated at room temperature in the dark for 15 min and subjected to flow cytometry analysis using BD FACS Canto II Flow Cytometer.

### Determination of the Activities of Antioxidant Enzymes

HeLa cells were collected and washed twice with PBS before lysis. The lysates were ultracentrifuged at 12,000 g for 30 min. The activities of antioxidant enzymes (SOD and GSH) were measured according to the manufacturer’s protocol (Kit S0109 and S0053, Beyotime Institute of Biotechnology, PR China).

### ROS Measurement

The ROS levels were measured by a ROS Assay Kit with DCFH-DA according to the manufacturer’s instructions. Briefly, after AE-treatment, the cell culture medium was replaced with a serum-free medium containing DCFH-DA (10 μM). After incubation at 37°C for 15 min, cells were washed three times with a serum-free cell culture medium to remove additional DCFH-DA. ROS scavenger NAC (5 mM) was pre-treated for 2 h. Fluorescence images were observed and captured under a fluorescence microscope.

### Immunofluorescence Labeling and Confocal Microscopy

Immunofluorescence labeling was conducted according to our standard protocols (Meng et al., 2020). At the indicated time post-AE-treatment, cells were fixed with 4% PFA for 10 min in PBS and permeabilized for 15 min with 0.2% Triton X-100 in PBS. After blockade of nonspecific binding by incubation of cells for 30 min with 2% bovine serum albumin (BSA) in PBS, samples were incubated with the appropriate primary antibodies and subsequently incubated with fluorescein-labeled secondary antibodies (ThermoFisher). Confocal fluorescence images were acquired at the confocal microscope (Olympus). All image data shown are representative of randomly selected fields from at least three replicates.

### SDS-PAGE and Immunoblotting

Western-Blot (WB) assay was conducted according to our standard protocols (Meng et al., 2020). Briefly, cell lysates were mixed with 5 × SDS loading buffer, boiled at 95°C for 5 min, and then subjected to SDS-PAGE. Proteins were transferred to PVDF membranes and subjected to the following steps. Membranes were blocked for 30 min by 5% nonfat milk in TBST and then incubated with primary antibody for 1 h at room temperature. After three washes with TBST, membranes were incubated with the HRP-conjugated second antibody for 30 min. After another three washes, membranes were incubated in the chemiluminescent substrate, and the antibody-bound protein was detected using LAS 4000 (Fujifilm). Mouse primary antibodies were diluted according to the manufacturer’s instructions when used in immunoblotting.

### Transcriptomic Analysis

HeLa cells were treated with AE (50 μM) for 6 or 40 h. Total RNA was extracted using TRIzol reagent, and subjected to RNA transcriptome sequencing after determination of its quality. RNA transcriptome sequencing was performed by Biomarker Technologies (Beijing, China) following the standard protocol. The reads were mapped to the reference genome of *Homo sapiens*. Relative gene expression was calculated in FPKM. Changes in more than twofold gene expression with *p* < 0.05 were considered reliable and statistically significant. Differential expressed genes (DEGs) were functionally classified using GO functional enrichment and KEGG pathway analysis using online analysis tools of DAVID (the Database for Annotation, Visualization, and Integrated Discovery) (https://david.ncifcrf.gov/).

### Quantification and Statistical Analysis

Results are presented as mean ± SD (standard deviation) containing at least three biological replicates. Data were analyzed using a Student’s *t*-test to compare two experimental groups. A difference is considered significant as the following: *p < 0.05, **p < 0.01.

## Results

### AE Inhibits Cell Growth, Cell Migration and Induces Cell Death in HeLa Cells by Activation of Caspase Cascade

**FIGURE 1:**
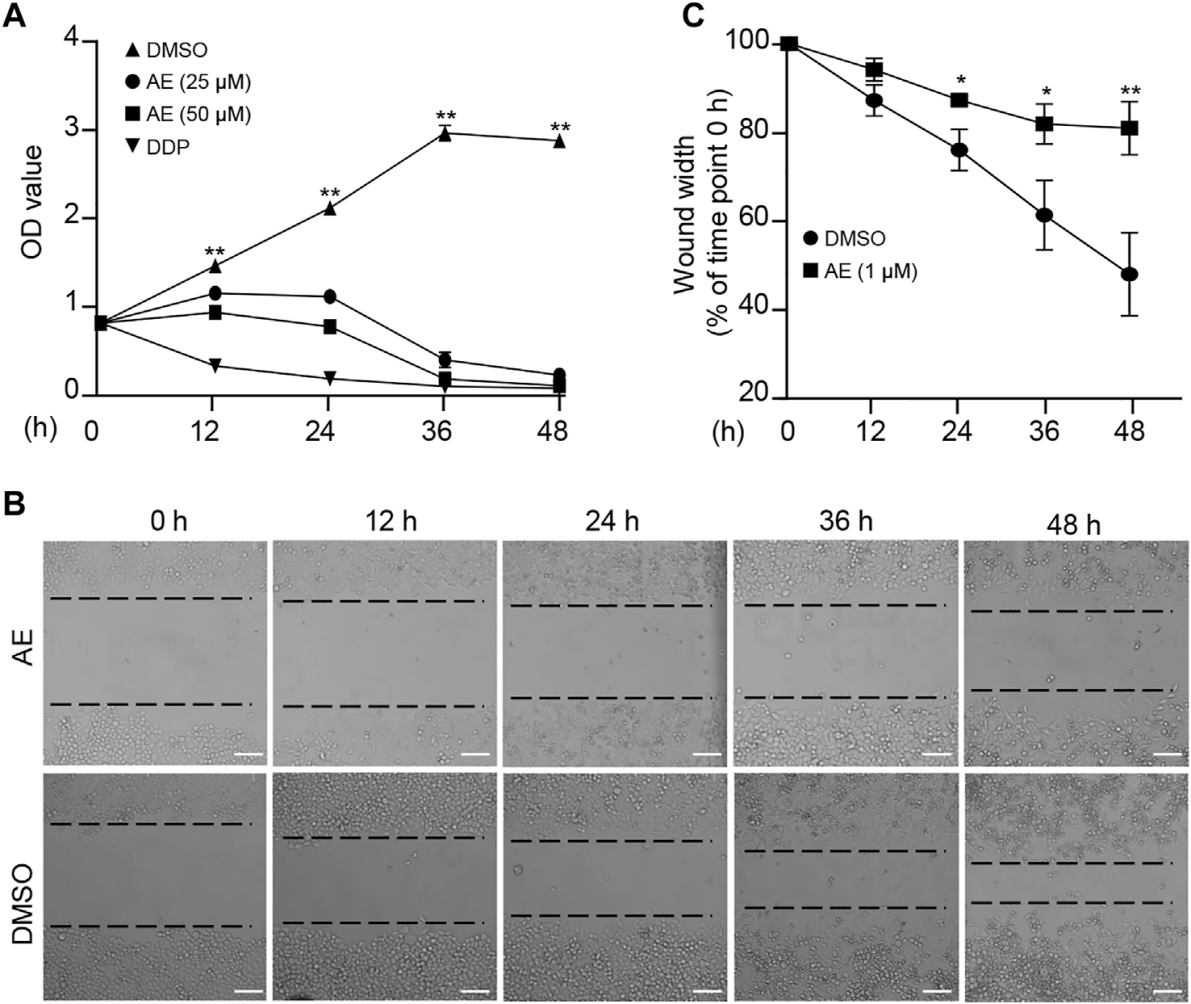
AE inhibits the proliferation and migration of HeLa cells. **(A)** Effects of AE on the proliferation of HeLa cells. HeLa cells were treated with AE or DDP for the indicated hours, and the cell proliferation was examined by the CCK-8 assay. **(B,C)** Effects of AE on the migration of HeLa cells. Cell migration was detected by wound scratch assay. The wound width was calculated using ImageJ. Results are as means ± SD from three independent experiments. *p < 0.05, **p < 0.01. Scale bar, 100 μm.

**FIGURE 2:**
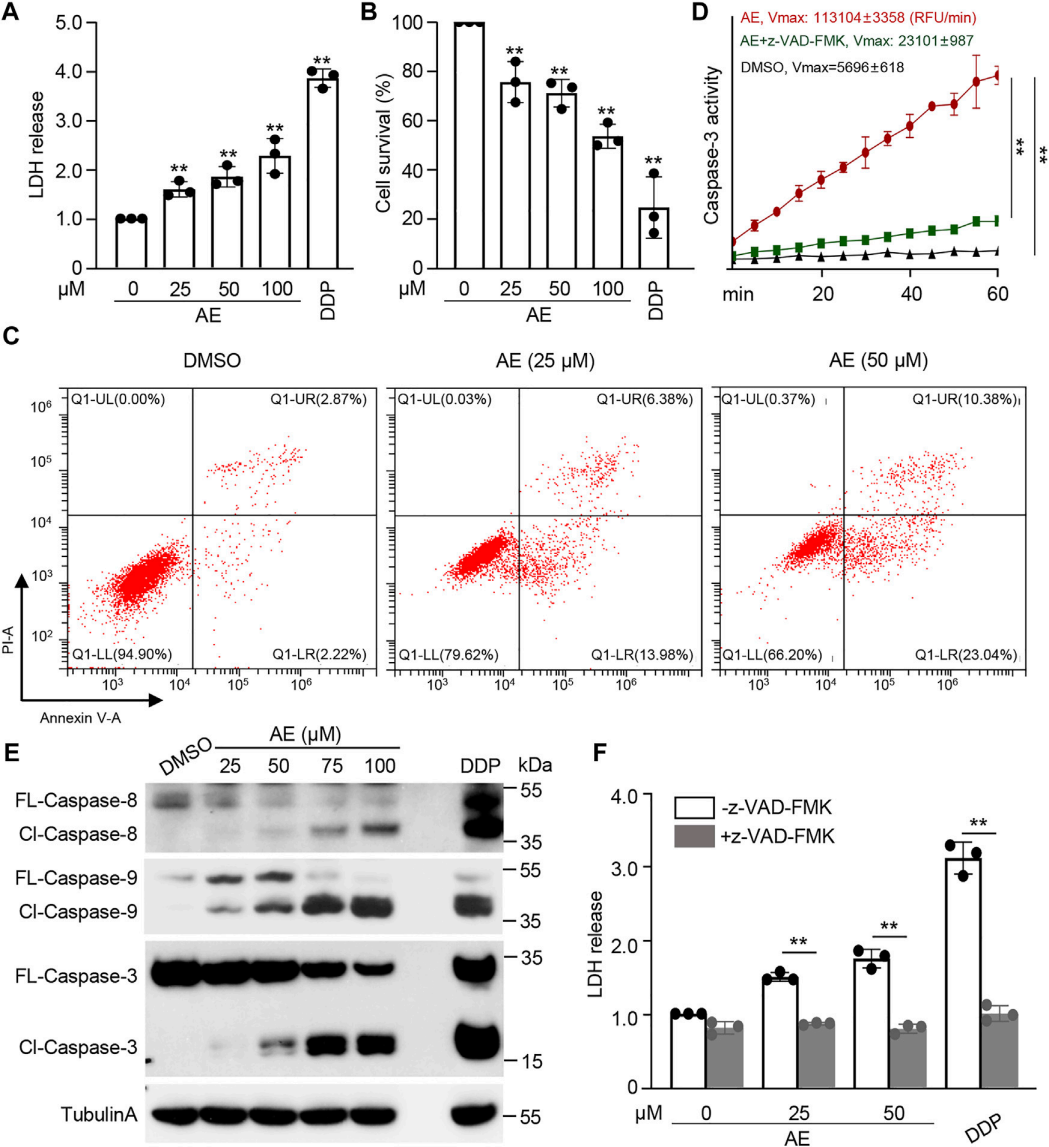
AE induces caspase-mediated cell death of HeLa cells. **(A–C)** Effects of AE on the cell death of HeLa cells. HeLa cells were treated with AE or DDP for 16 h. Cell death was examined by the released LDH **(A)**, cellular ATP **(B)**, and flow cytometry with annexin V/PI staining **(C)**. **(D,E)** Effects of AE on the caspase activation of HeLa cells. HeLa cells were treated with AE or DDP for 24 h. Cell lysates were prepared and subjected to caspase-3 activity assay **(D)** and immunoblotting analysis with the indicated caspase antibodies **(E)**. Caspase activity was calculated as relative fluorescence units (RFU) changes per minute (Vmax). **(F)** Inhibition of caspase activity reverses the AE mediated-cell death of HeLa cells. HeLa cells were treated with AE or DDP for 24 h, and the cell death was examined by the released LDH. Caspase inhibitor z-VAD-FMK was added at 2 h before AE or DDP treatment. Data in C and E are representative of three independent experiments. The statistical data are expressed as means ± SD from three independent experiments. **p < 0.01.

We first examine the effects of AE on the characteristics of cancer cells. The chemotherapy drug cisplatin (DDP) was set as a positive control. CCK8 assays showed significant inhibition of HeLa proliferation in a dose-manner at concentrations following AE treatment of 25 and 50 μM (Figure 1A). Also, in the presence of AE, HeLa cells migrated slower into the scratch area than control at 24, 36, and 48 h (Figure 1B). The relative wound width at each time point was calculated by comparing with that at 0 h (Figure 1C). Meanwhile, AE treatment induced significant cell death based on detection of the LDH release (Figure 2A) and reduced the cell viability based on detection of ATP contents in a dose-dependent manner (Figure 2B). The CC_50_ for AE was 30.09 μM, which was determined with cell viability data (Supplementary Figure S1). Annexin V-FITC/PI assay showed that AE could induce phosphatidylserine exposure and plasma membrane permeabilization of HeLa cells, indicating cell death (Figure 2C).

To examine whether AE activates the caspase cascade, enzymatic activities against synthetic caspase-3/7 substrate Ac-DEVD-AFC were assayed. Caspase activity was significantly higher in HeLa cells treated by AE compared to the control group (Figure 2D). Immunoblotting analyses showed that both initiator caspases (caspase-8/9) and effector caspase (caspase-3) were activated and cleaved to their cleaved forms in AE-treated cells in a dose-dependent manner (Figure 2E). Pretreatment of a pan-caspase inhibitor z-VAD-FMK completely blocked AE-induced caspase activity (Figure 2D) and cell death (Figure 2F). Thus, AE-induced cell death of HeLa cells is caspase-dependent.

### AE Triggers GSDME-Dependent Pyroptosis in HeLa Cells

**FIGURE 3:**
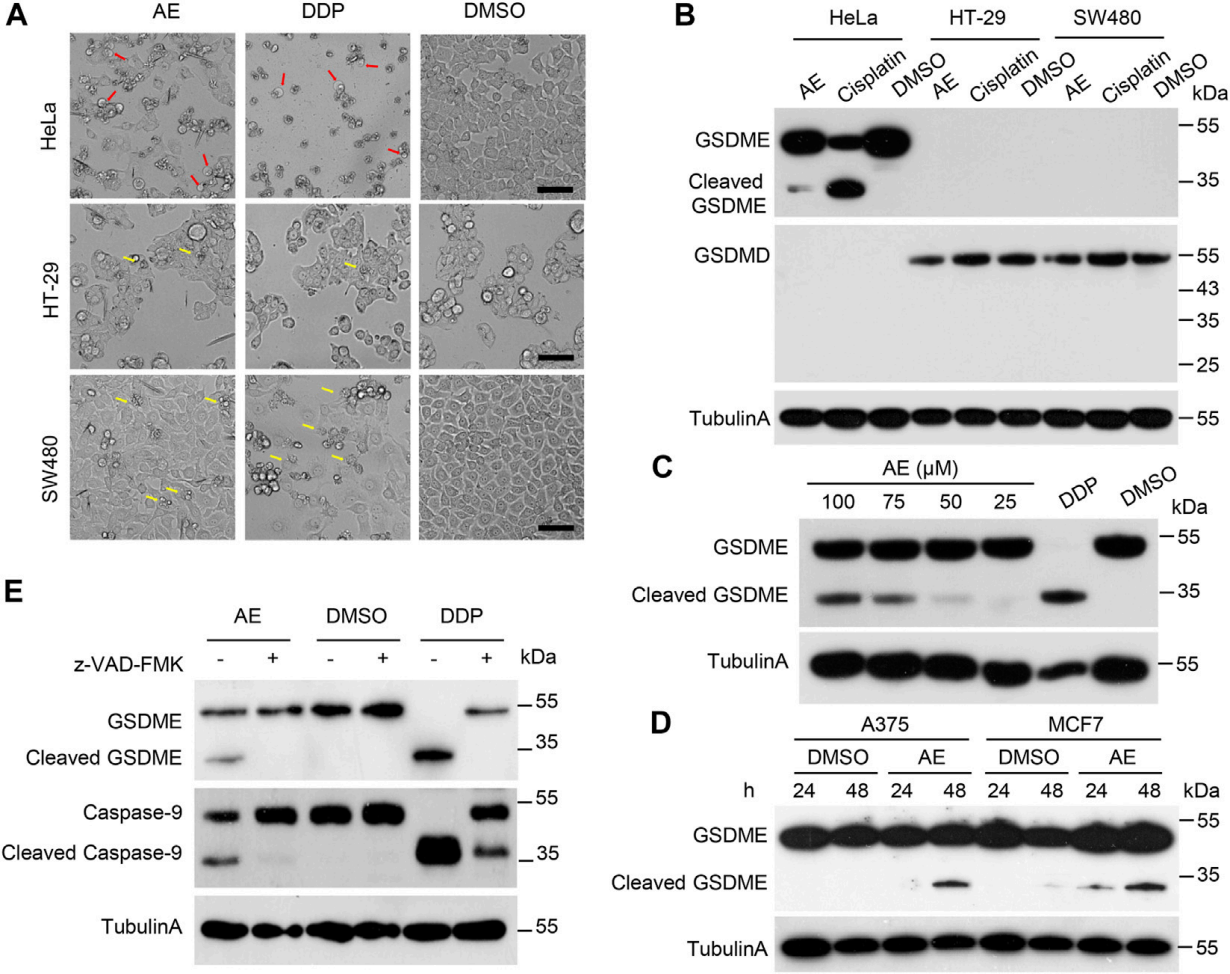
AE activated the caspase9/caspase3/GSMDE axis and induced pyroptosis of HeLa cells. **(A)** The effects of AE on the morphology of HeLa cells. HeLa, HT-29, and SW480 cells were treated with AE (50 μM) or DDP (5 mg/L) for 16 h. Shown are representative microscopic images of cells. The red arrows indicate the pyroptotic cells, and the yellow arrows represent the apoptotic cells. Scale bar, 100 μm. **(B)** Immunoblotting detection of GSDMs cleavage in AE-treated HeLa cells. Cell lysates in **(A)** were prepared and subjected to immunoblotting analysis with the indicated antibodies. **(C)** Immunoblotting detection of GSDMs cleavage in HeLa cells in AE-dosed manner. **(D)** Immunoblotting detection of GSDME cleavage in AE-treated A375 and MCF7 cells. Cells were cultured with AE (50 μM) for 24 or 48 h, followed by immunoblotting analysis with the indicated antibodies. **(E)** Inhibition of caspase activity reverses the AE mediated-GSDME cleavage. HeLa cells were treated with AE (50 μM) or DDP (5 mg/L) for 24 h, followed by immunoblotting analysis. Caspase inhibitor z-VAD-FMK was added at 2 h before AE or DDP treatment. Data of immunoblots are representative of three independent experiments.

Next, we investigated the mechanisms underlying AE-induced HeLa cell death. The microscope images showed that HeLa cells treated with AE and DDP became round and swollen with bubble-like structures from the plasma membrane, representing the typical pyroptosis morphology (Figure 3A, red arrows). To determine which gasdermin mediates this process, cleavages of GSDMD and GSDME were examined. Immunoblotting results showed that GSDMD was not expressed or expressed at a low level in HeLa cells (Figure 3B). GSDME cleavage was observed in the presence of AE in a dosed manner (Figure 3C), indicating that GSDME rather than GSDMD is activated in AE-treated HeLa cells. Two additional pieces of evidence supported these results. First, in human colon cancer cell lines (SW480 and HT-29) that naturally expressed GSDMD but no other gasdermins, AE only triggered apoptotic cell death (Figure 3A, yellow arrows). Second, AE induced the GSDME activation in GSDME-expressed cells (A375 and MCF7 cells) (Figure 3D). Thus, we concluded that AE could cause pyroptosis dependent on GSDME. Additionally, AE treatment activated caspase-9 and caspase-3 (Figure 2E), and pretreatment of z-VAD-FMK attenuated the cleavages of caspase-9 and GSDME (Figure 3E), suggesting that the caspase-9/3/GSDME axis might activate AE-induced pyroptosis.

To further support the key conclusions that AE induces pyroptosis dependent on GSDME in HeLa cells, we synthesized three pairs of siRNA and found that the three# pair had the best down-regulation effect. As expected, when *gsdme* was knockdown, the percentage of pyroptotic cells induced by AE significantly decreased, and the percentage of apoptosis increased (Supplementary Figure S2). In addition, we tested the GSDME expression in several cell lines maintained in our lab, and selected 293T cells for the GSDME over-expression experiment due to the lack of endogenous GSDME and its high transfection efficiency. As expected, overexpression of GSDME led to pyroptosis when treated with AE, accompanied by the GSDME cleavage (Supplementary Figure S3).

### Effects of the Hydroxyanthraquinone Derivatives on GSDME Activation

**FIGURE 4:**
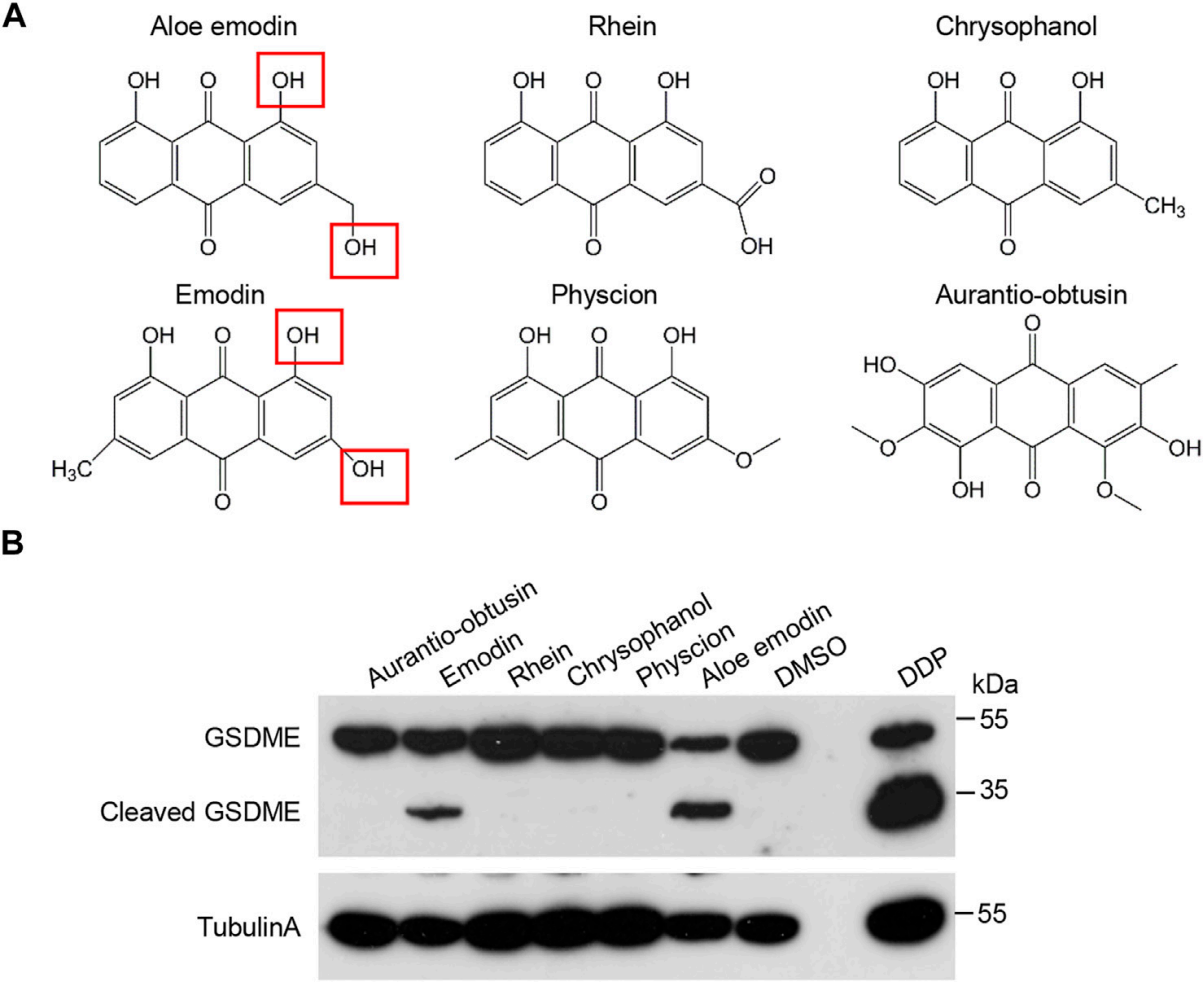
Effects of the hydroxyanthraquinone derivations on GSDME activation. **(A)** The schematic structures of hydroxyanthraquinone derivations that are similar to AE. **(B)** Effects of the hydroxyanthraquinone derivations in **(A)** on the GSDME cleavage. HeLa cells were treated with 50 μM of chrysophanol, emodin, aurantio-obtusin, physcion, aloe-emodin, or rhein for 16 h, followed by immunoblotting analysis with the indicated antibodies. Data of immunoblots are representative of three independent experiments.

Given that Aloe-emodin shares a very similar structure with rhubarb anthraquinone aglycones (Figure 4A), we investigated whether these drugs are also involved in GSDME activation in HeLa cells. Among them, apparent cleavage of GSDME was detected upon Emodin and AE treatment but not in other compounds, including Rhein, Chrysophanol, Aurantio-obtusin, and Physcion (Figure 4B).

### AE Induces Mitochondrial Dysfunction

**FIGURE 5:**
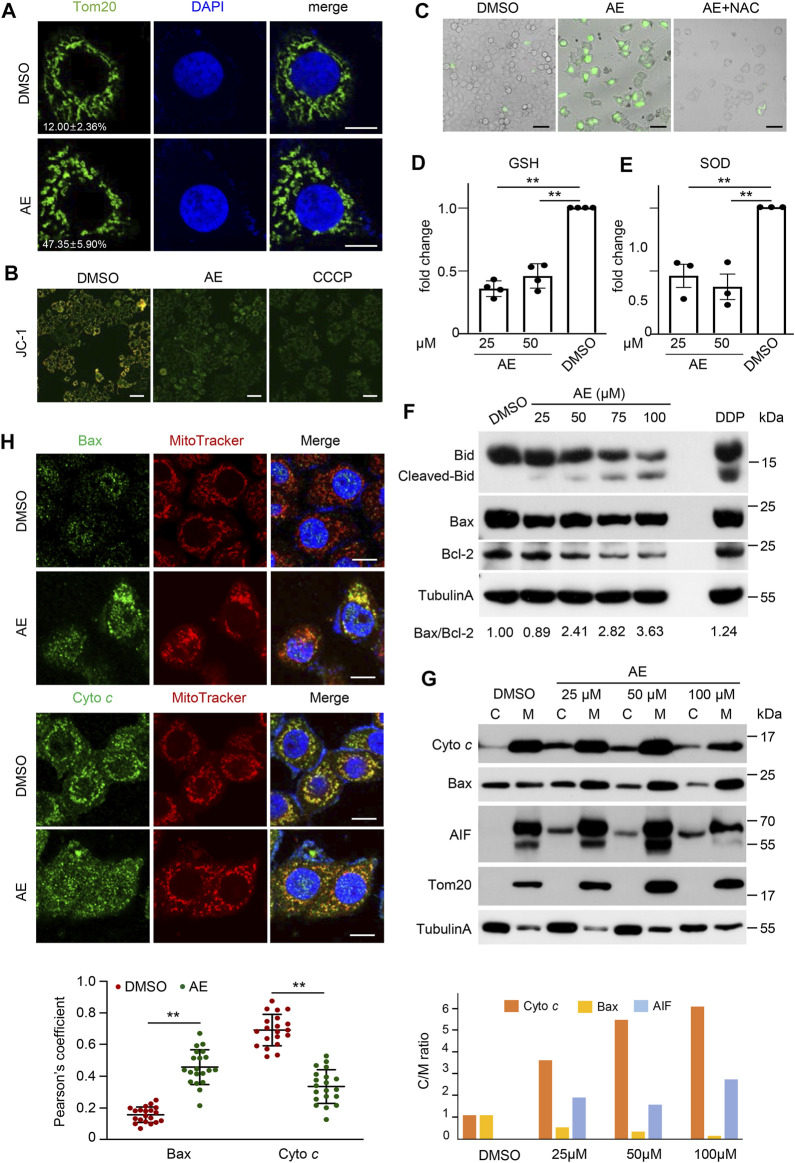
AE induces mitochondrial dysfunction of HeLa cells. **(A)** Effects of AE on the mitochondrial morphology. HeLa cells were treated with AE (50 μM) for 24 h. Fluorescence detection of Tom20 (green) and DAPI (blue) are shown. Statistics of cells showing obvious mitochondrial ministacks are listed in the lower-left corner. At least 100 cells were counted for each group, and the statistical data shown are from three independent determinations. Scale bar, 10 μm. **(B)** Effects of AE on the mitochondrial membrane potential. The mitochondrial membrane potential was determined by the JC-1 assay. The merged fluorescence detections of JC-1 aggregates (red) and JC-1 monomer (green) are shown. Color change from orange to green indicated that the JC-1 changed from aggregates to monomers, and that the mitochondrial membrane potential changed from a high level to a low level. Scale bar, 100 μm. **(C–E)** Effects of AE on the mitochondrialoxidativestress. HeLa cells were treated with 50 μM of AE for 24 h. ROS was detected by H2DCF-DA staining **(C)**, and antioxidant enzymes levels of GSH **(D)** and SOD **(E)** were measured. ROS scavenger NAC (5 mM) was pre-treated for 2 h. **(F)** Effects of AE on the expression of Bcl-2 family proteins. HeLa cells were treated with AE or DDP for 24 h, followed by immunoblotting analysis. Bax/Bcl-2 ratio was calculated by comparing the band intensities of Bax protein to Bcl-2 protein using the ImageJ software. **(G,H)** Effects of AE on the cellular translocation of mitochondrial proteins. Cells were treated with AE for 24 h. **(G)** Total membrane and cytosol proteins were isolated and immunoblotted with the corresponding antibodies. M: membrane fraction. **(C)** cytoplasmic fraction. **(H)** Colocalization of cytochrome c or BAX (green) with MitoTracker (red) is shown in fluorescence images (upper) and the statistics of Pearson correlation coefficient (lower). The Pearson correlation coefficient was calculated from more than 20 cells for each experiment using ImageJ software. Vertical lines represent SD. **p < 0.01. Scale bar, 25 μm. Data are representative of three independent experiments.

The mitochondrial pathway is involved in various stimuli, including chemotherapeutic drugs, TNF treatment, and UV radiation (Lin and Beal, 2006; Canta et al., 2015). Prompted by this notion, we examined mitochondrial functions in cells treated with AE. AE treatment in HeLa cells broken down the mitochondrial ribbon into dispersed ministacks. Mitochondrial structures were stained by the anti-Tom20 antibody and MitoTracker, respectively (Figures 5A,H). Simultaneously, the JC-1 staining assay indicated that AE caused induced mitochondrial membrane potential (ΔΨm) depletion in HeLa cells (Figure 5B). In addition, AE exposure might increase mitochondrial oxidative stress, with the increased intracellular ROS generation and decreased antioxidant enzymes glutathione (GSH) and superoxide dismutase (SOD) levels (Figures 5C–E).

Mitochondrial disruption during cell death is regulated by the apoptosis-inducing factor (AIF) and Bcl-2 family proteins, which results in the release of cytochrome *c* (cyto *c*) that directly interacts with Apaf-1 and caspase-9 to form the apoptosomes. In apoptosomes, caspase-9 is activated by dimerization, which initiates the mitochondrial pathway (Gulbins et al., 2003). To determine the molecular events underlying AE-induced mitochondrial dysfunction during cell death, we examined the expression and translocation of these proteins. Immunoblotting results showed that AE increased the Bid cleavage and Bax/Bcl-2 ratio in a dosed manner (Figure 5F). AE also led to Bax translocation to membrane parts and caused cyto *c* and AIF partitioning into the cytosol phase (Figure 5G). Besides, endogenous BAX formed a punctate perinuclear structure and showed a clear colocalization with the mitochondria upon AE-treatment (Figure 5H). In contrast, cyto *c* diffused in the cytoplasm and showed less mitochondrial localization (Figure 5H). Therefore, AE induces mitochondrial dysfunction and promotes Bid/Bax-induced cytochrome *c* release.

### Systematically Transcriptional Analysis of AE-Treated HeLa Cells

**FIGURE 6:**
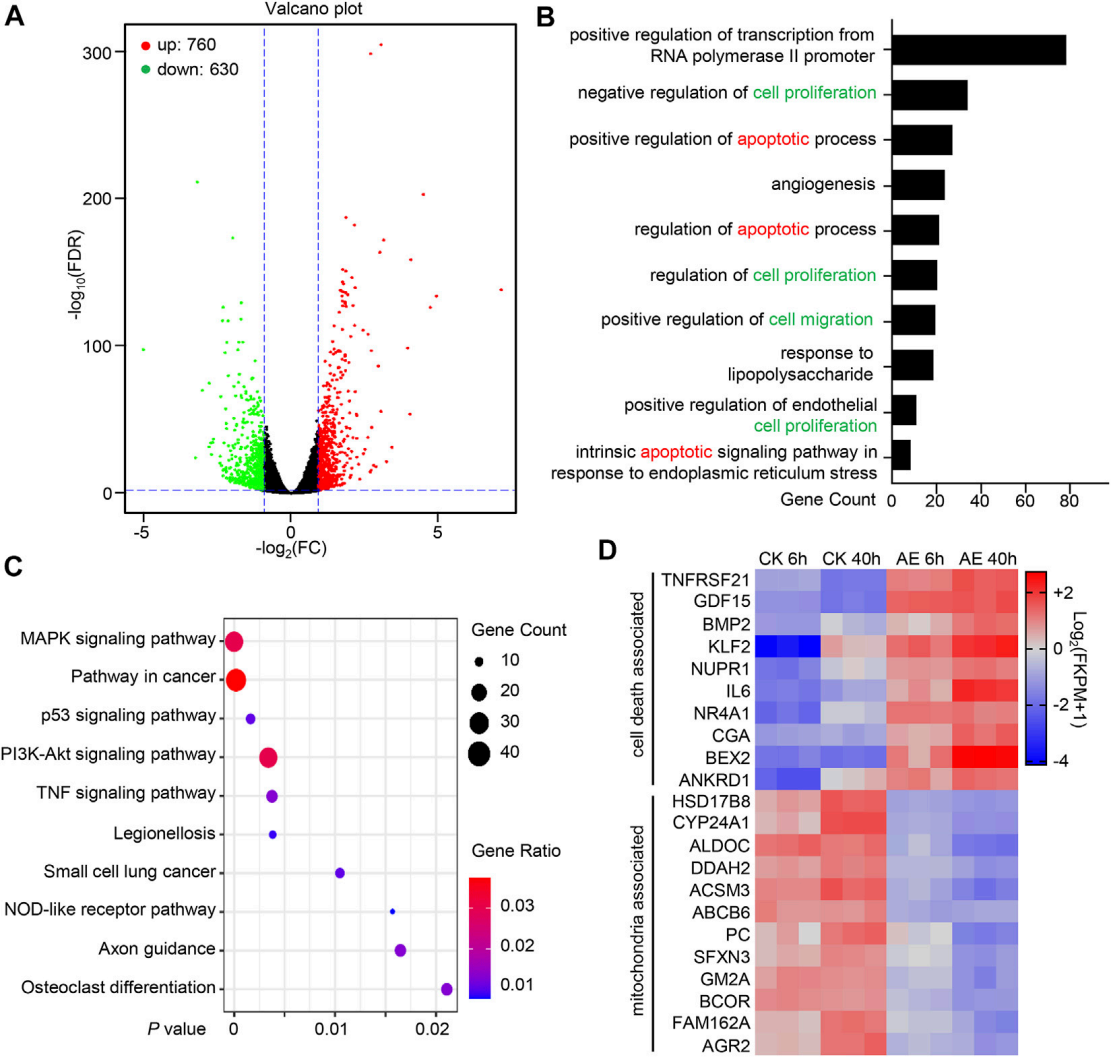
Systematically Transcriptional Analysis of AE-treated HeLa cells. **(A)** Volcano map shows DEGs’ overall scatter in HeLa cells at 6 h post-treatment. Each point represents a specific gene transcript. The abscissa indicates the fold-change of gene expression in AE-treated samples divided by those in control samples. The ordinate shows the statistical test values for the significant difference in expression. Red dots correspond to up-regulated genes, green dots refer to the significantly down-regulated genes, and black dots indicate a non-significant change in gene expression. **(B)** Gene Ontology (GO) enrichment analysis of the DEGs into biological processes using the DAVID (the Database for Annotation, Visualization, and Integrated Discovery) online analysis tool (https://david.ncifcrf.gov/). The lower abscissa indicates the number of genes annotated to a specific GO term. **(C)** Kyoto Encyclopedia of Genes and Genomes (KEGG) pathway enrichment analysis of the DEGs using the DAVID online analysis tool. The horizontal axis represents the *p-value*. The big circle indicates a more significant number of DEGs enriched in this function. **(D)** Heatmap shows the synergistic expression patterns of the DEGs involved in regulating cell death or mitochondrial function at 6 h or 40 h post-AE treatment. Color change (from blue to red) indicated that the relative intensity changed from low to high.

To provide a comprehensive understanding of the effects of AE treatment in gene expression and cell signaling pathways in epithelial HeLa cells, a transcriptomic analysis was conducted. Considering that HeLa cells exhibited apparent pyroptosis morphology at approximately 24 h post-AE-treatment (Figure 3A), we selected 6 and 40 h, representing the early and late phases, respectively. At 6 h, 1,390 differentially expressed genes (DEGs) were identified in the aloe-emodin treatment group, among which 760 genes were up-regulated and 630 were down-regulated (Figure 6A). A total of 976 DEGs were mapped to GO terms, and the top 10 enriched GO functions were listed. The functions were mainly divided into positive regulation of transcription, inflammation response, cell proliferation, and cell death process (Figure 6B). In addition, a total of 405 DEGs were subjected to KEGG pathway analysis, and ten mostly predominant pathways were identified. These pathways were related to the MAPK signaling pathway, pathway in cancer, p53 signaling pathway, PI3K-Akt signaling pathway, and TNF signaling pathway (Figure 6C). Considering that genes expression was transient and different at early and later stages, we compared DEGs at 6 and 40 h post AE treatment. A total of 91 genes were synergistically expressed, among which 36 genes were up-regulated, and 56 were down-regulated. Interestingly, 10 upregulated genes were involved in the cell death process, and 12 downregulated genes were related to mitochondrial functions (Figure 6D), which was consistent with our functional study above. Thus, transcriptome analysis provides genome-wide gene expression changes during the treatment of AE.

## Discussion

**FIGURE 7:**
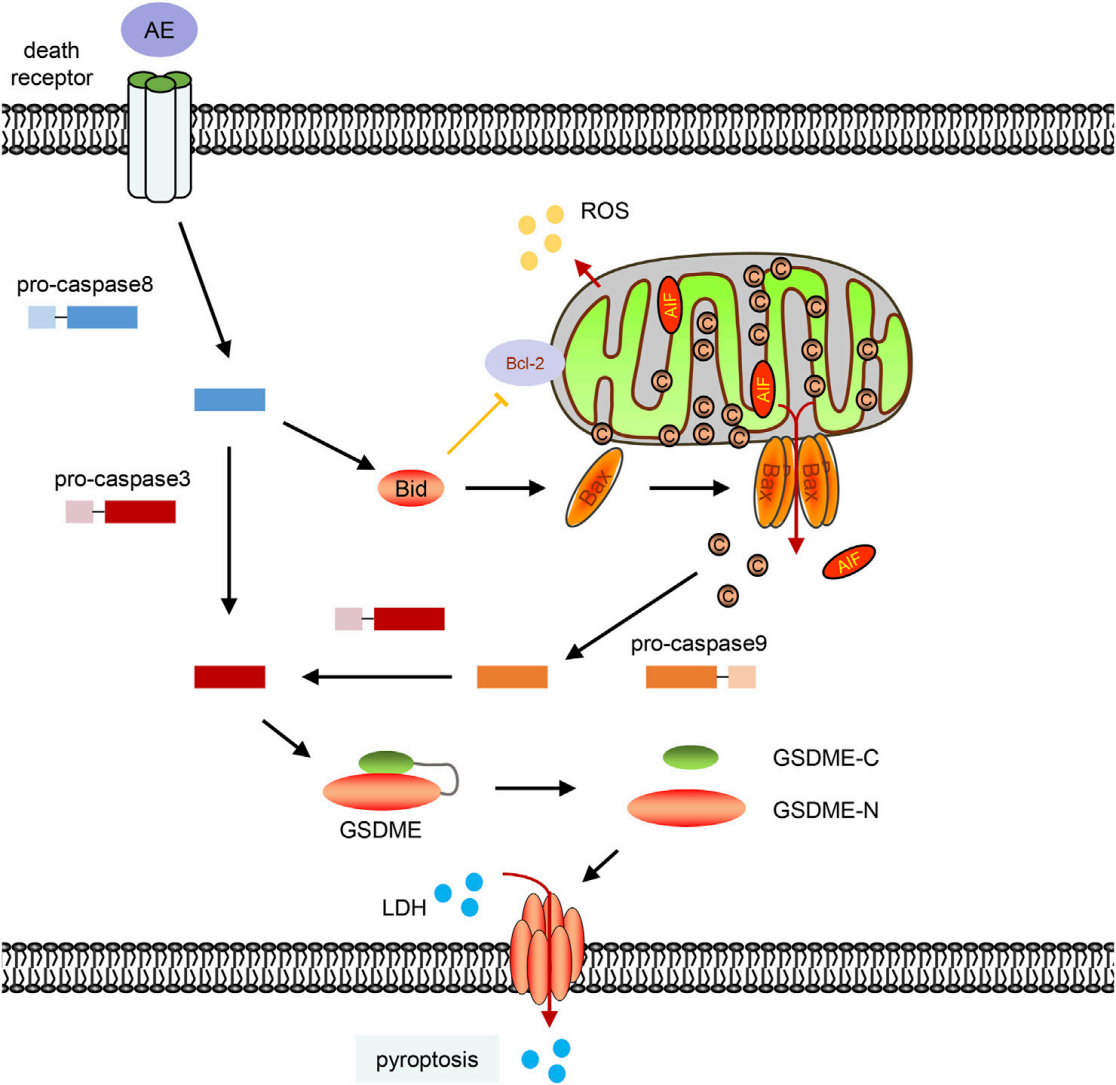
A schematic diagram of this work. Aloe-emodin can induce caspase-8 activation, Bid cleavage, BAX translocation to permeabilize the mitochondrial membrane and release cytochrome c into the cytosol. Cytochrome c then activates caspase-9 and subsequent caspase-3. Active caspase-3 mediates the GSDME cleavage, and GSDME-N could directly oligomerize and cause plasma membrane lysis which will cause pyroptosis characteristics, including LDH release and the formation of plasma membrane bubbles.

Aloe-emodin exhibits a broad spectrum of pharmacological benefits, such as anticancer, anti-inflammatory, antivirus, antibacterial activities (Dong et al., 2020). Of most importance, AE shows remarkable anticancer effects in lung, breast, colon, pancreatic cancer cells by inducing apoptosis and inhibiting cancer cell proliferation (Sanders et al., 2018). Mechanically, AE affects the MAPKs, PKC, Ras/ERK, ROS-JNK, PI3K/Akt/mTOR pathway (Acevedo-Duncan et al., 2004; Tu et al., 2016; Tseng et al., 2017; Dou et al., 2019; Shen et al., 2020), and regulates the expression of a set of genes, such as the casein kinase II, ALP, c-Myc, ER*α*, NAT, and NF-κB (Chen et al., 2014; Dong et al., 2020). To our knowledge, we first discovered that AE could trigger pyroptosis by inducing mitochondrial dysfunction and activating the Bax/caspase9/caspase3/GSDME pathway in HeLa cells (Figure 7). In addition, we provide a systematically transcriptional analysis of pathways and gene expression in AE-treated cells.

Both activations of GSDMD and GSDME could induce pyroptotic cell death. However, it seems that GSDME, rather than GSDMD, contributed to AE-induced pyroptosis because SW480 cell, which has a high level of GSDMD but without GSDME expression, was refractory to AE-caused pyroptosis. On the contrary, in GSDME-expressing cells such as A375 and MCF7 cells, GSDME was cleaved by caspase-3 cleaves, and the pore-forming ability of the N-terminus was released. Therefore, AE may trigger pyroptosis in GSDME high expression cells but apoptosis in GSDME-deficient cells.

Aloe-emodin could induce caspase activation via the death receptor pathway (caspase-8 activation) and the mitochondrial pathway (caspase-9 activation) (Figure 2E). Suppression of caspase-8 inhibited AE-induced the cyto *c* release and caspase-9 activation (Lin et al., 2010), indicating that the death receptor pathway controlled the AE-induced mitochondrial dysfunction. In consistent with this, we also observed that AE induced Bid cleavage (Figure 5F). Since activation of caspase-8 in the death receptor pathway results in cleavage of Bid, and translocation of activated Bid activates mitochondria pathway (Yin, 2000; Kim et al., 2017), AE-induced mitochondrial dysfunction is then subsequently linked via cleavage of Bid to the death receptor pathway (Figure 7).

Conventional chemotherapy effectively inhibits tumor growth, but the tumor becomes insensitive to chemotherapy in the later stage, and many patients relapse over time (Norden et al., 2008). Chemotherapy resistance is one of the significant problems for effective clinical therapy. In etoposide-resistant melanoma cells, loss of GSDME decreased cell response to etoposide. In contrast, over-expression of GSDME increased the cell sensitivity to etoposide, suggesting that increased GSDME activation is related to reduced etoposide resistance (Lage et al., 2001). Interestingly, a combination of AE or the *Aloe vera* extract increased the cellular sensitivity to chemotherapeuticagents and was more effective in killing cancer cells (Luo et al., 2014). Still, the mechanism of this action is largely unknown. Our results showed that AE activates the caspase-9/caspase-3/GSDME axis. However, it is worth noting that the ability of AE to induce pyroptosis is much lower than that of DDP, because AE-induced pyroptosis may require a higher concentration or more treatment time (Figure 3D). In consistent with this, PLK1 inhibitor BI2536 can increase cisplatin chemosensitivity by accelerating GSMDE-mediated pyroptosis, but BI2536 treatment alone only causes GSMDE activation to a much less extent (Wu M. et al., 2019). Thus, these findings may explain potential roles in reversing chemotherapyresistance in GSDME-expressed cancer cells.

Several anthraquinones derivatives are found in the well-known Chinese herbal medicines, and have been developed as pharmacological tools and drugs (Malik and Müller, 2016; Diaz-Munoz et al., 2018). Besides AE, we found that emodin-treatment could also trigger GSDME cleavage. By preliminary comparison of the chemical structures of the six anthraquinones in this study, we found that AE and emodin share similar free hydroxyl groups at positions 1 and 3 (Figure 4), which may be the reason for GSDME cleavage. However, it needs to be confirmed using more derivatives. Thus, it is intriguing to investigate whether other anthraquinone derivatives also induce pyroptosis and find out which anthraquinones have the most potent effects or whether/how they could interact with each other. Thus, this study reveals a novel pharmacological characteristic of anthraquinone derivatives, which provides valuable information for the potential use of anthraquinone containing Chinese herbs.

We here showed that AE could kill GSDME-expressed cancer cells by pyroptotic cell death, making it a potent anti-cancer agent. In addition, GSDME-mediated pyroptosis of tumour cells enhances the it phagocytosis by tumour-associated macrophages, and triggers the recruitment of immune cells to induce anti-tumor inflammatory responses (Zhang et al., 2020; Li et al., 2021). However, it should be noted that GSDME is expressed in various normal tissues, including immune system cells (www.biogps.org). Thus, AE-mediated pyroptosis may induce toxicity and cause disorder in immune system in certain normal human cells. There have been negative effects reported on AE, such as hepatotoxicity and nephrotoxicity (Zhu et al., 2012; Dong et al., 2017; Dong et al., 2020). Besides, because AE is difficult to be absorbed by the small intestine and has a short half-life (Yu et al., 2016), it has not been used clinically extensively. Thus, researches are urged to enhance its oral bioavailability, improve tumor-targeting property, and reduce the toxicity to normal cells (Şeker Karatoprak et al., 2022). One *in vitro* study reported that AE-loaded in SBA-15 demonstrated better water solubility, and exhibited particular toxicity on HeLa cells and little effect on the normal cervical cells (Jangra et al., 2021), but extensive *in vivo* researches are required before its clinical implications.

Taken together, here we found that AE induces mitochondrial dysfunction and activates the Bax-caspase9-caspase3-GSDME axis. AE exerts pyroptosis in the GSDME-expressed tumor cells. Besides, AE treatment causes extensive changes in gene expressions and cellular pathways. These results of this study suggest a novel mechanism for anthraquinone derivatives in the treatment of cancer cells.

## Data Availability

The raw data of transcriptome presented in the study are deposited in the Genome Sequence Archive in BIG Data Center, Beijing Institute of Genomics (BIG) repository, accession number PRJCA007478 that are publicly accessible at https://bigd.big.ac.cn/gsa-human/browse/HRA001640. The source data supporting the findings of this study can be found in the article/Supplementary Material.

## Abbreviations

AE, Aloe-emodin; AIF, apoptosis-inducing factor; ATCC, American Type Culture Collection; BSA, bovine serum albumin; CCK-8, cell counting kit-8; Cyt c, cytochrome c; DAVID, the Database for Annotation, Visualization and Integrated Discovery; DDP, cisplatin; DEG, differentially expressed gene; DMSO, dimethyl sulfoxide; DMEM, Dulbecco’s modified Eagle’s medium; GSDM, gasdermin; GSH, glutathione; ΔΨm, mitochondrial membrane potential; GO, Gene Ontology; KEGG, Kyoto Encyclopedia of Genes and Genomes; LDH, lactate dehydrogenase; PFA, paraformaldehyde; PBS, phosphate-buffered saline; RFU, relative fluorescence unit; RCD, regulated cell death; SOD, superoxide dismutase; TNF, tumor necrosis factor.
