# Supplementary material for: Aloe-Emodin Induces Mitochondrial Dysfunction and Pyroptosis by Activation of the Caspase-9/3/Gasdermin E Axis in HeLa Cells
Source: Front Pharmacol. 2022 May 17;13:854526. doi: 10.3389/fphar.2022.854526 (PMC9157280; doi:10.3389/fphar.2022.854526)
Supplement: Supplementary file 1 [file Presentation1.PPTX]

## Slide 1
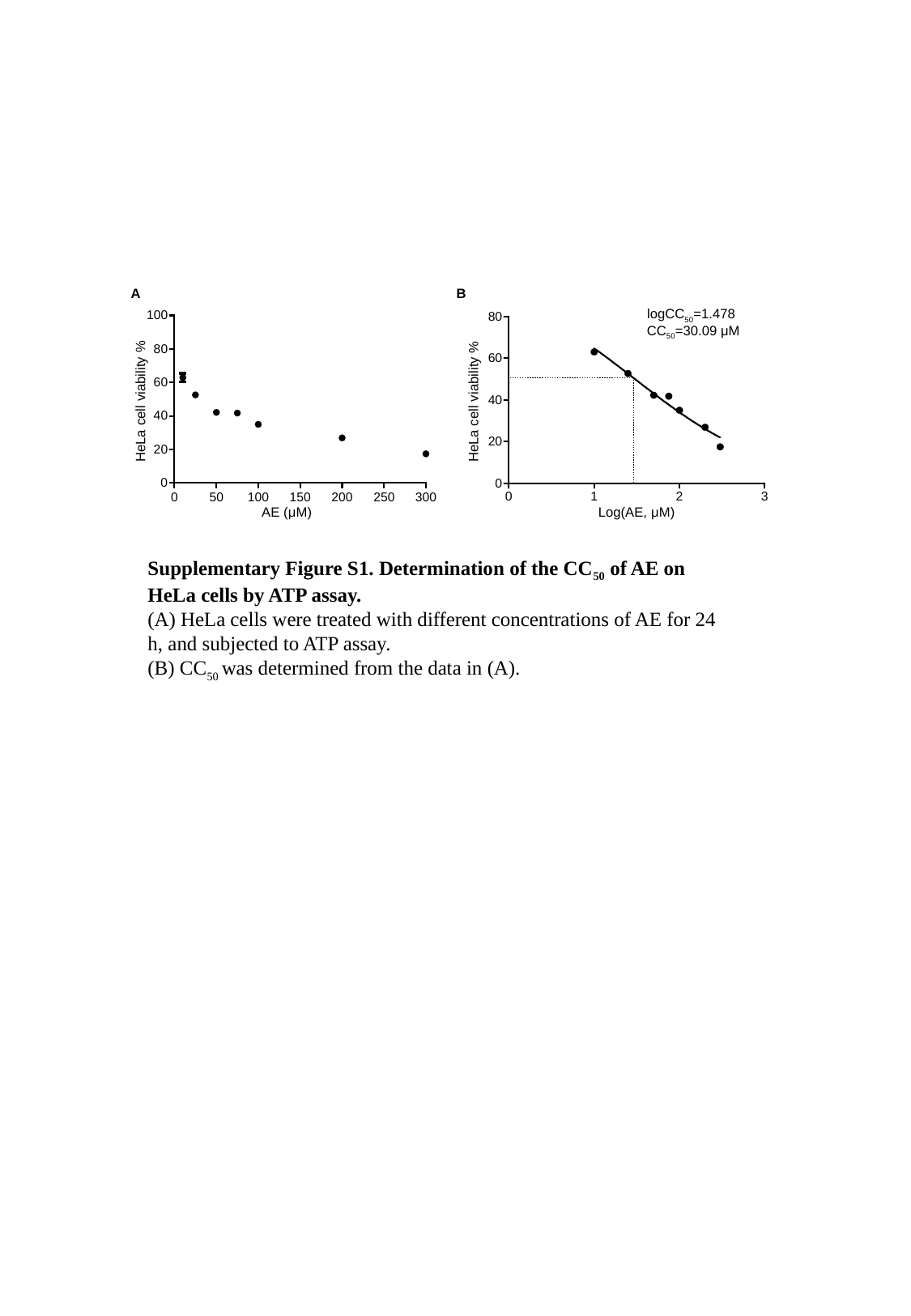

A
B
HeLa cell viability %
Log(AE, μM)
logCC50=1.478
CC50=30.09 μM
HeLa cell viability %
AE (μM)
Supplementary Figure S1. Determination of the CC50 of AE on HeLa cells by ATP assay.
(A) HeLa cells were treated with different concentrations of AE for 24 h, and subjected to ATP assay.
(B) CC50 was determined from the data in (A).

## Slide 2
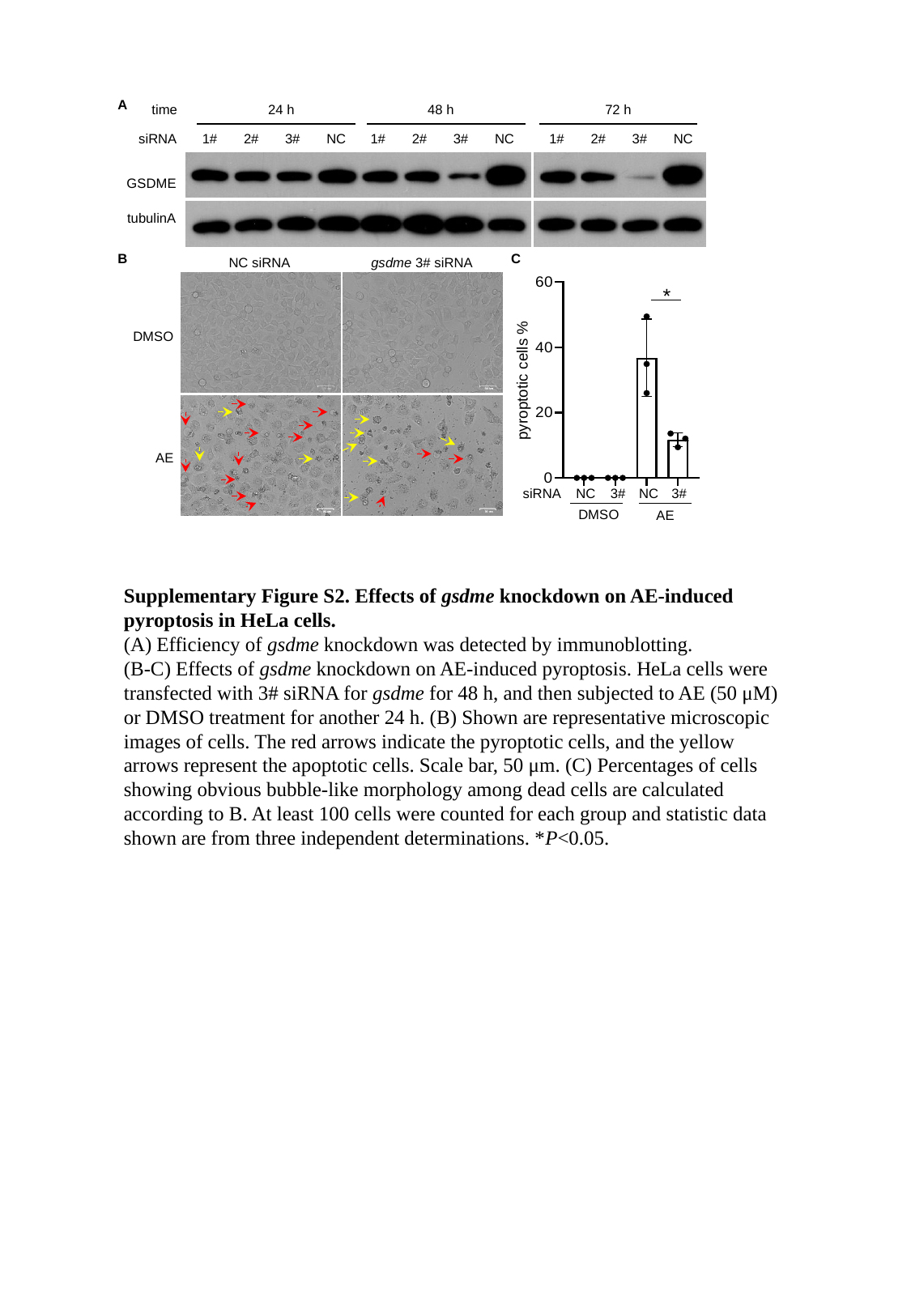

A
time
24 h
48 h
72 h
siRNA
1#
2#
3#
NC
1#
2#
3#
NC
1#
2#
3#
NC
GSDME
tubulinA
B
C
NC siRNA
gsdme 3# siRNA
DMSO
AE
*
pyroptotic cells %
siRNA
NC
3#
NC
3#
DMSO
AE
Supplementary Figure S2. Effects of gsdme knockdown on AE-induced pyroptosis in HeLa cells.
(A) Efficiency of gsdme knockdown was detected by immunoblotting.
(B-C) Effects of gsdme knockdown on AE-induced pyroptosis. HeLa cells were transfected with 3# siRNA for gsdme for 48 h, and then subjected to AE (50 μM) or DMSO treatment for another 24 h. (B) Shown are representative microscopic images of cells. The red arrows indicate the pyroptotic cells, and the yellow arrows represent the apoptotic cells. Scale bar, 50 μm. (C) Percentages of cells showing obvious bubble-like morphology among dead cells are calculated according to B. At least 100 cells were counted for each group and statistic data shown are from three independent determinations. *P<0.05.

## Slide 3
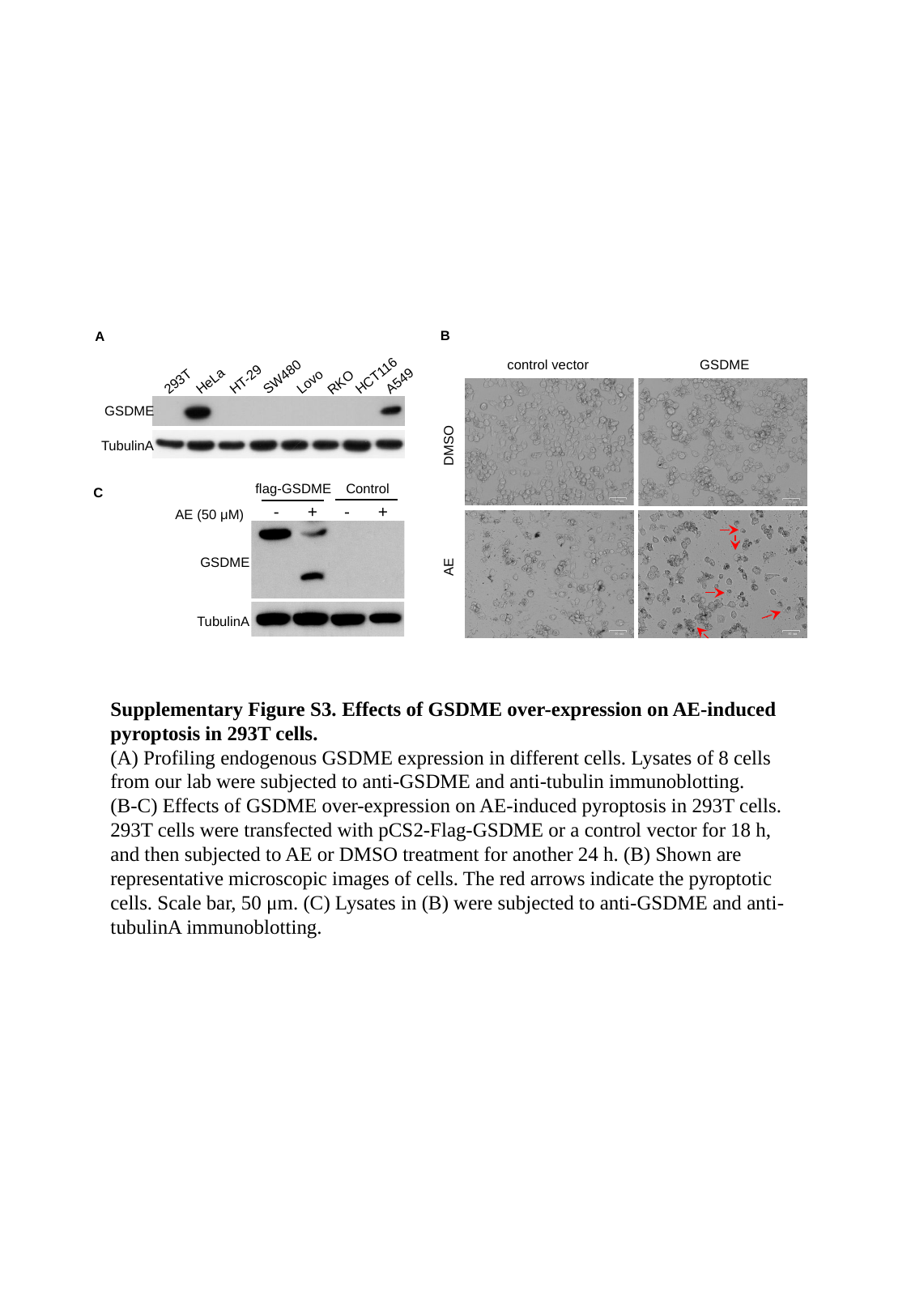

B
A
control vector
GSDME
DMSO
AE
293T
SW480
HCT116
Lovo
HT-29
A549
HeLa
RKO
GSDME
TubulinA
flag-GSDME
Control
C
-
+
-
+
AE (50 μM)
GSDME
TubulinA
Supplementary Figure S3. Effects of GSDME over-expression on AE-induced pyroptosis in 293T cells.
(A) Profiling endogenous GSDME expression in different cells. Lysates of 8 cells from our lab were subjected to anti-GSDME and anti-tubulin immunoblotting.
(B-C) Effects of GSDME over-expression on AE-induced pyroptosis in 293T cells. 293T cells were transfected with pCS2-Flag-GSDME or a control vector for 18 h, and then subjected to AE or DMSO treatment for another 24 h. (B) Shown are representative microscopic images of cells. The red arrows indicate the pyroptotic cells. Scale bar, 50 μm. (C) Lysates in (B) were subjected to anti-GSDME and anti-tubulinA immunoblotting.
